# Prognostic nomogram for overall survival in pediatric osteosarcoma with pulmonary metastases: a SEER database analysis

**DOI:** 10.3389/fped.2025.1574034

**Published:** 2025-04-17

**Authors:** Jiaxiang Tang, Yun Guo, Hongting Lu, Yifan Fang, Weiming Chen

**Affiliations:** ^1^ Department of Pediatric Surgery, Fujian Children’s Hospital (Fujian Branch of Shanghai Children’s Medical Center), College of Clinical Medicine for Obstetrics & Gynecology and Pediatrics, Fujian Medical University, Fuzhou, China; ^2^ Department of Pediatric Surgery, Women and Children’s Hospital Affiliated to Qingdao University, Qingdao, China

**Keywords:** osteosarcoma, pulmonary metastasis, prognosis, nomogram, pediatric osteosarcoma

## Abstract

**Background:**

Pulmonary metastasis (PM) is the most common site of distant metastasis in osteosarcoma (OS), particularly in pediatric cases, which are associated with poor prognosis. However, limited research has focused on identifying prognostic factors (PFs) for pediatric osteosarcoma with pulmonary metastasis (POPM). This study aims to identify clinical features and PFs of POPM and develop a validated nomogram to predict overall survival in POPM patients.

**Methods:**

A retrospective analysis was conducted using OS cases from the Surveillance, Epidemiology, and End Results (SEER) database (2010–2021). Clinical characteristics were compared between patients with and without PM. PFs were identified using Least Absolute Shrinkage and Selection Operator (LASSO) regression and evaluated through Kaplan–Meier analysis. Patients were divided into training (*N* = 148) and validation (*N* = 64) cohorts. Independent PFs were determined via Cox regression to construct a prognostic nomogram, which was assessed using the concordance index (C-index), the area under the receiver operating characteristic curve (AUC-ROC), and calibration plots. Decision curve analysis (DCA) was used to evaluate clinical applicability.

**Results:**

LASSO regression identified key PFs: AJCC stage, T stage, median household income, systemic therapy, and time from diagnosis to treatment. Among these, all except T stage were validated as independent PFs via Cox regression. The nomogram demonstrated strong predictive accuracy with C-index values of 0.68 (training) and 0.71 (validation). AUC values for 1-, 3-, and 5-year survival were 0.786, 0.709, and 0.711 in the training cohort and 0.780, 0.760, and 0.776 in the validation cohort. Calibration plots showed excellent concordance between predicted and actual survival, and DCA confirmed the nomogram's clinical relevance.

**Conclusion:**

AJCC stage, median household income, systemic therapy, and time from diagnosis to treatment are significant PFs for POPM survival. The validated nomogram provides a valuable tool for personalized prognostic assessment and treatment decision-making in clinical practice.

## 1 Introduction

Osteosarcoma (OS) is an aggressive primary bone tumor that originates from the uncontrolled proliferation of mesenchymal cells responsible for osteoid matrix production. It is a prevalent bone cancer that mainly affects adolescents and children during periods of rapid skeletal growth (1, 2). Although rare, with an incidence of approximately 2–3 cases per million annually, OS accounts for 0.2% of all malignant tumors and 11.7% of primary bone tumors. The incidence is higher in males, with a male-to-female ratio of approximately 1.4:1 (2). OS is characterized by a high mortality rate and poor prognosis, largely due to its potential to metastasize, particularly to the lungs, early in the disease course (3, 4).

Recent advancements in neoadjuvant chemotherapy and surgical techniques have significantly improved outcomes for patients with localized disease, with 5-year survival rates now reaching 60%–70%. However, survival rates for patients with distant metastases remain poor, at approximately 20%–30% (5–7). Pulmonary metastasis (PM) is the most common site of distant dissemination in OS, representing a major therapeutic challenge and contributing significantly to treatment failure. Surgical resection of PM remains essential for improving survival and achieving potential cures in affected patients (8). Therefore, accurate prognostic evaluation is essential for informing personalized treatment strategies and guiding clinical decision-making.

Previous studies have identified several prognostic factors (PFs) in OS, including age, histological subtype, tumor size and location, surgical resectability, response to neoadjuvant chemotherapy, and presence of metastasis at diagnosis (9–11). However, there is a lack of large-scale, population-based studies focusing specifically on pediatric osteosarcoma with pulmonary metastasis (POPM). Most available data are derived from small, single-center retrospective studies, limiting their generalizability and statistical power required for conclusive inferences (12, 13). The identification and prediction of PFs for POPM remain challenging due to the multifactorial nature of disease progression and survival outcomes in pediatric oncology. Nomograms are reliable statistical tools that integrate multiple PFs into a visual format, allowing for individualized and accurate prognostic predictions (10, 14). This study used data from the Surveillance, Epidemiology, and End Results (SEER) database (2010–2021) to investigate clinical features and PFs in POPM. A prognostic nomogram was developed and validated to predict overall survival in this population. To the best of our understanding, this is the largest retrospective study to evaluate survival and PFs in pediatric OS patients with PM.

## 2 Materials and methods

### 2.1 Patient selection and study design

Data for this study were obtained from the SEER database, a comprehensive, population-based cancer registry comprising 18 registries and covering approximately 28% of the U.S. population (15). Managed by the National Cancer Institute, the SEER database includes extensive demographic and clinical information, including sex, age, race, tumor characteristics, American Joint Committee on Cancer (AJCC) stage, systemic therapy, metastasis status, and survival outcomes. Since 2010, the database has also included site-specific metastasis information (e.g., lung, liver, bone, brain), facilitating the analysis of POPM. Given the public and de-identified nature of the SEER data, ethical approval and informed consent were not required following the Declaration of Helsinki.

Patients were included based on the following criteria: (1) diagnosis of osteosarcoma as defined by the International Classification of Childhood Cancer (ICCC) site recode (3rd edition)/International Agency for Research on Cancer (IARC) 2017 classification; (2) diagnosis between 2010 and 2021; (3) age at diagnosis between 0 and 19 years; and (4) complete follow-up data. Exclusion criteria included: (1) the presence of multiple primary malignancies; and (2) missing data on race, AJCC stage, T stage, surgical status, time from diagnosis to treatment (days), or metastasis status (lung and bone). A total of 1,162 pediatric OS cases met the inclusion criteria, of which 212 presented with PM. These 212 POPM cases were randomly assigned to training (*N* = 148) and validation (*N* = 64) cohorts using a 7:3 ratio. Overall survival was defined as the time from diagnosis to death or last follow-up.

### 2.2 Nomogram model development and confirmation

To identify potential PFs for POPM, the Least Absolute Shrinkage and Selection Operator (LASSO) regression algorithm was applied to the entire cohort of POPM cases. LASSO regression improves model performance by penalizing overfitting and selecting key variables with high predictive value. This method offers advantages over traditional stepwise Cox regression by addressing multicollinearity and reducing model complexity. Following LASSO analysis, univariate and multivariate Cox proportional hazards regression analyses were conducted in the training group to identify independent PFs associated with overall survival. A prognostic nomogram was then constructed using the significant variables from multivariate analysis to predict 1-, 3-, and 5-year overall survival in POPM patients. The model's predictive performance was evaluated using the concordance index (C-index), the area under the receiver operating characteristic curve (AUC-ROC), and calibration plots. Decision curve analysis (DCA) was used to assess clinical efficacy. Internal validation of the nomogram was conducted using 1,000 bootstrap resampling iterations.

### 2.3 Statistical analysis

**Figure 1:**
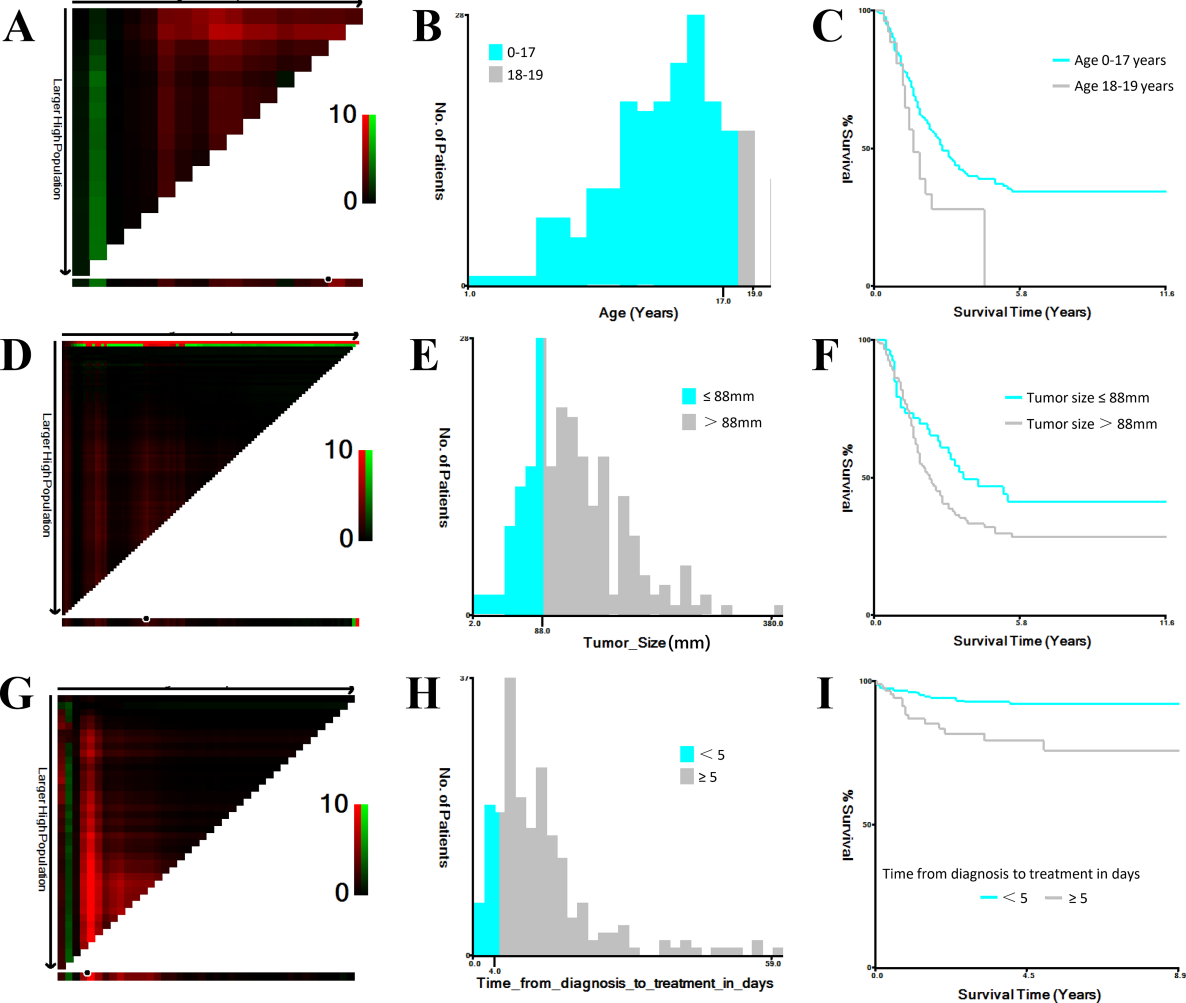
Optimal cutoff values for age, tumor size, and time from diagnosis to treatment identified using X-tile. **(A,B)** Determination of optimal age cutoff values. **(C)** Kaplan–Meier survival curves for age subgroups (0–17 years and 18–19 years). **(D,E)** Determination of optimal tumor size cutoff values. **(F)** Kaplan–Meier survival curves for tumor size subgroups (<8.8 cm and ≥8.8 cm). **(G,H)** Determination of optimal cutoff values for time from diagnosis to treatment (days). **(I)** Kaplan–Meier survival curves for time-to-treatment subgroups (<5 days and ≥5 days).

All statistical analyses were performed using SPSS (version 26.0; IBM Corp., Armonk, NY, USA) and R software (version 4.3.0; https://www.R-project.org). The following R packages were used: glmnet 4.1–8 for LASSO regression with 10-fold cross-validation, survival 3.8–3 for Cox regression modeling, rms 7.0–0 for nomogram construction, and timeROC 0.4 for time-dependent ROC curve analysis. Continuous variables were presented as medians with interquartile ranges, while categorical variables were summarized as frequencies and percentages. The X-tile program (Yale University, New Haven, CT, USA) was used to determine optimal cutoff points for continuous variables (Figure 1). Age was categorized as 0–17 years and 18–19 years; tumor size thresholds were set at <8.8 cm and ≥8.8 cm; and time from diagnosis to treatment was grouped as <5 days and ≥5 days. Group comparisons for categorical variables were performed using chi-square or Fisher's exact tests, while continuous variables were compared using *t*-tests or Mann–Whitney *U* tests, as appropriate. Kaplan–Meier survival curves were generated, and log-rank tests were used to assess differences in survival. A *p* value <0.05 was considered statistically significant.

## 3 Results

### 3.1 Clinical features

**Figure 2:**
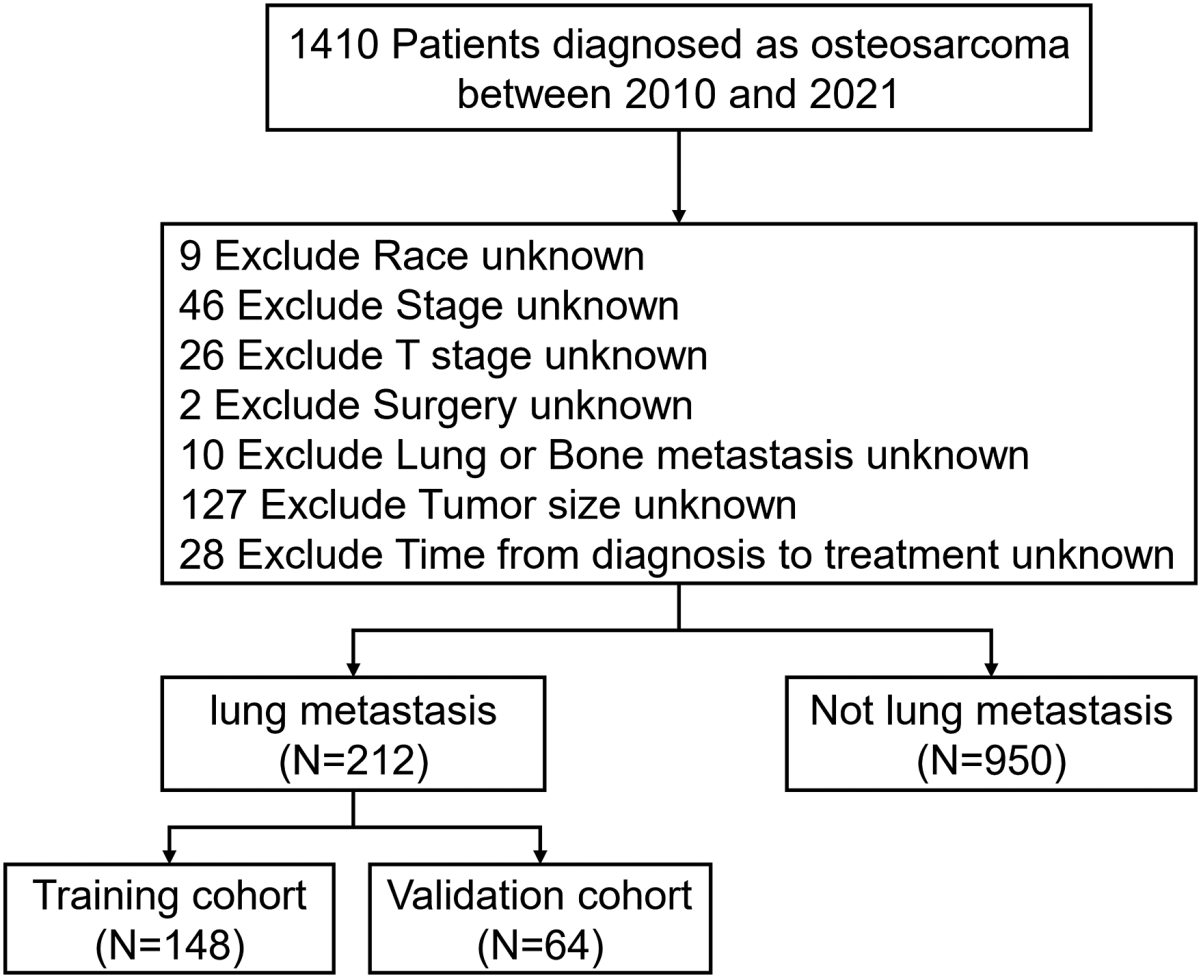
Flowchart illustrating patient selection based on inclusion and exclusion criteria.

A total of 1,410 pediatric OS patients were identified in the SEER database from 2010 to 2021. After applying the inclusion and exclusion criteria, 1,162 patients were included in the final analysis. Among them, 212 patients (18.24%) presented with PM at diagnosis, while 950 patients (81.76%) did not (Figure 2).

Comparative analysis of clinical features between patients with and without PM revealed statistically significant differences in several variables, including time from diagnosis to treatment (days), tumor size, overall survival, T stage, N stage, type of surgery, surgical intervention, radiation therapy, chemotherapy, systemic therapy, and vital status (*p* < 0.05). However, no significant differences were observed in age, sex, ethnicity, median household income, or tumor site (*p* > 0.05). Detailed results are summarized in Table 1.

**Table 1:** Comparison of prognostic factors between osteosarcoma patients with and without pulmonary metastasis.

Variables	Total (*n* = 1,162)	No (*n* = 950)	Yes (*n* = 212)	Statistic	*P*
Age, years, M (Q_1_, Q_3_)	14.00 (11.00, 16.00)	13.00 (11.00, 16.00)	14.00 (11.00, 16.00)	*Z* = −0.68	0.499
Time from diagnosis to treatment in days, M (Q_1_, Q_3_)	12.00 (7.00, 19.00)	13.00 (7.00, 20.00)	11.00 (7.00, 16.00)	*Z* = −2.63	0.009
Tumor Size, mm, M (Q_1_, Q_3_)	90.00 (67.00, 130.75)	87.00 (65.00, 124.00)	118.00 (85.00, 160.00)	*Z* = −7.08	<.001
Overall survival, month, M (Q_1_, Q_3_)	42.50 (19.00, 88.00)	49.00 (22.00, 92.00)	21.00 (11.75, 43.25)	*Z* = −7.95	<.001
Sex, *n* (%)				*χ*^2^ = 3.27	0.071
Female	492 (42.34)	414 (43.58)	78 (36.79)		
Male	670 (57.66)	536 (56.42)	134 (63.21)		
Race, *n* (%)				*χ*^2^ = 0.14	0.933
Black	174 (14.97)	144 (15.16)	30 (14.15)		
Other	136 (11.70)	111 (11.68)	25 (11.79)		
White	852 (73.32)	695 (73.16)	157 (74.06)		
Ethnicity, *n* (%)				*χ*^2^ = 0.75	0.387
Non-Spanish	791 (68.07)	652 (68.63)	139 (65.57)		
Spanish	371 (31.93)	298 (31.37)	73 (34.43)		
Median household income, *n* (%)				*χ*^2^ = 1.51	0.471
<$70,000	353 (30.38)	296 (31.16)	57 (26.89)		
$70,000–$84,999	410 (35.28)	332 (34.95)	78 (36.79)		
$85,000+	399 (34.34)	322 (33.89)	77 (36.32)		
Site, *n* (%)				*χ*^2^ = 0.02	0.885
LBLL	876 (75.39)	717 (75.47)	159 (75.00)		
Other	286 (24.61)	233 (24.53)	53 (25.00)		
T stage, *n* (%)				*χ*^2^ = 55.93	<.001
T1	424 (36.49)	385 (40.53)	39 (18.40)		
T2	683 (58.78)	535 (56.32)	148 (69.81)		
T3/T4/TX	55 (4.73)	30 (3.16)	25 (11.79)		
N stage, *n* (%)				*χ*^2^ = 41.40	<.001
N0	1,098 (94.49)	917 (96.53)	181 (85.38)		
N1/NX	64 (5.51)	33 (3.47)	31 (14.62)		
Type of operation, *n* (%)				*χ*^2^ = 77.22	<.001
Amputation of limb	230 (19.79)	163 (17.16)	67 (31.60)		
Local tumor destruction or excision or unknown	98 (8.43)	89 (9.37)	10 (4.72)		
No surgery	71 (6.11)	37 (3.89)	34 (16.04)		
Radical excision or resection of lesion WITH limb salvage	762 (65.58)	661 (69.58)	101 (47.64)		
Surgery, *n* (%)				*χ*^2^ = 44.55	<.001
No	71 (6.11)	37 (3.89)	34 (16.04)		
Yes	1,091 (93.89)	913 (96.11)	178 (83.96)		
Radiation, *n*(%)				*χ*^2^ = 28.02	<.001
None/Unknown	1,119 (96.30)	928 (97.68)	191 (90.09)		
Yes	43 (3.70)	22 (2.32)	21 (9.91)		
Chemotherapy, *n* (%)				*χ*^2^ = 11.42	<.001
No/Unknown	49 (4.22)	49 (5.16)	0 (0.00)		
Yes	1,113 (95.78)	901 (94.84)	212 (100.00)		
Systemic therapy, *n* (%)				*χ*^2^ = 5.52	0.019
No	114 (9.81)	84 (8.84)	30 (14.15)		
Yes	1,048 (90.19)	866 (91.16)	182 (85.85)		
Status, *n* (%)				*χ*^2^ = 88.04	<.001
Alive	823 (70.83)	729 (76.74)	94 (44.34)		
Dead	339 (29.17)	221 (23.26)	118 (55.66)		

*Z*, Mann–Whitney test; *χ*^2^, chi-square test; M, median; Q_1_, 1st quartile; Q_3_, 3st quartile; LBLL: The long bones of lower limb.

### 3.2 Prognostic factors for POPM

**Figure 3:**
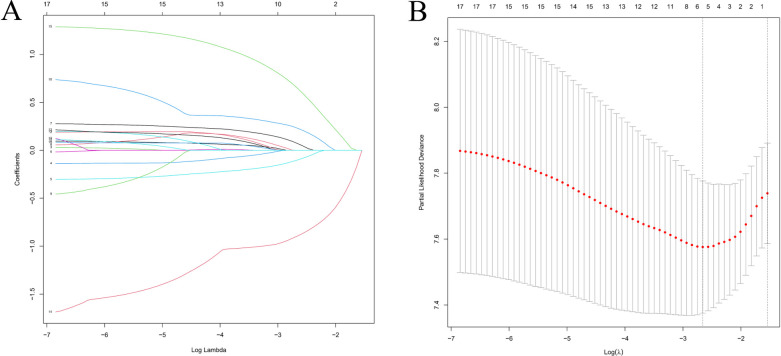
Feature selection using LASSO regression analysis. **(A)** Coefficient profiles of clinical variables plotted against Log(*λ*), where each colored line represents a variable. **(B)** Ten-fold cross-validation for tuning parameter selection. The vertical dashed line indicates the value of *λ* corresponding to the minimum partial likelihood deviance, representing the optimal model fit.

LASSO regression was applied to reduce multicollinearity among variables (Figure 3A). The optimal tuning parameter (lambda, *λ*) was determined via 10-fold cross-validation based on the minimum partial likelihood deviance. Figure 3A presents the trajectories of regression coefficients (*β*) for each variable as a function of Log(*λ*), while Figure 3B shows the partial likelihood deviance curve plotted against Log(*λ*), where lower values indicate better model fit. At the optimal *λ* corresponding to the minimum deviance, five variables with non-zero coefficients were identified as potential PFs: AJCC stage, T stage, median household income, systemic therapy, and time from diagnosis to treatment (days).

**Figure 4:**
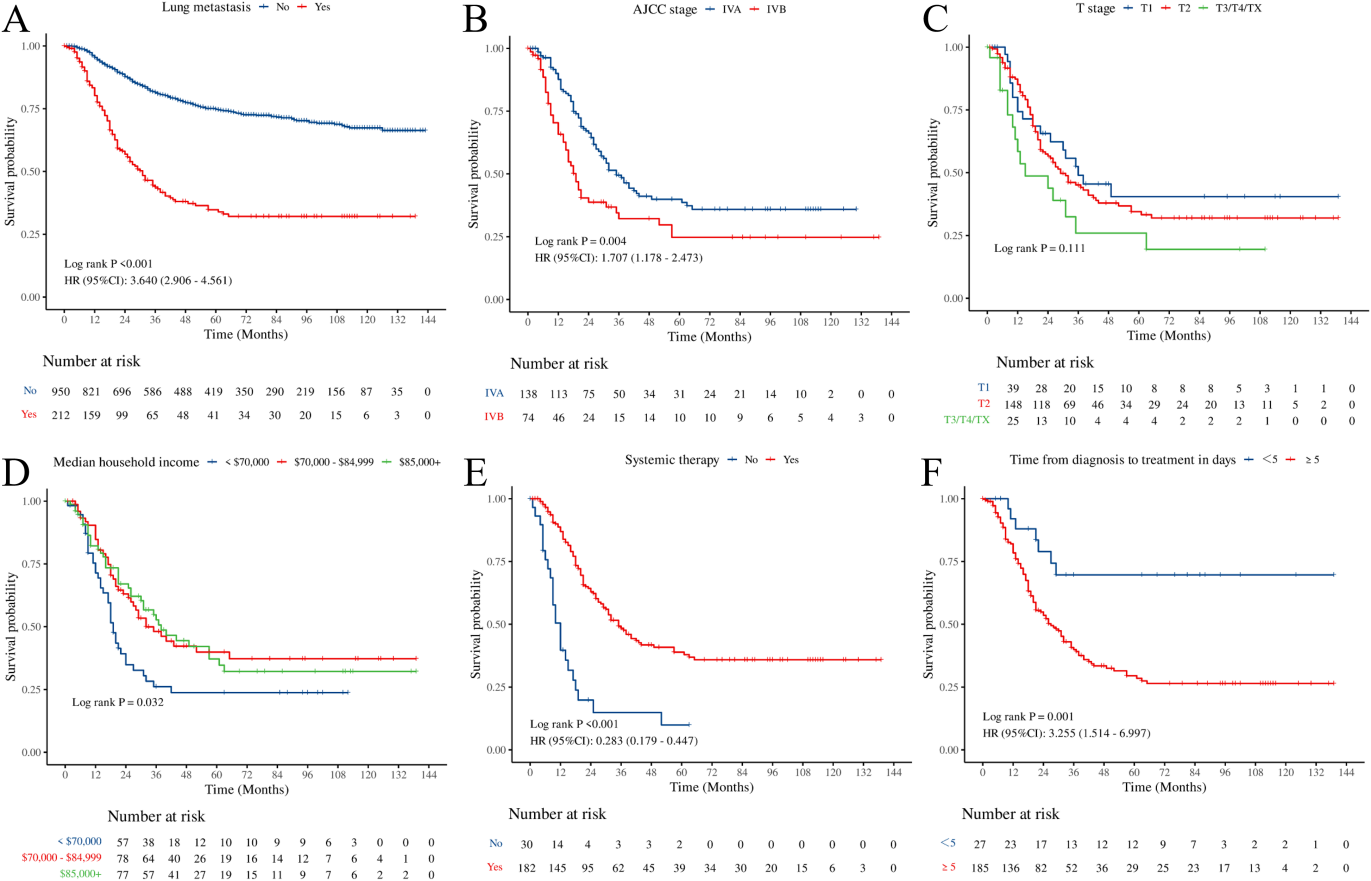
Kaplan–Meier curves illustrating overall survival. **(A)** Lung metastasis. **(B)** AJCC stage. **(C)** T stage. **(D)** Median household income. **(E)** Systemic therapy. **(F)** time from diagnosis to treatment in days.

Kaplan–Meier analysis demonstrated that pediatric OS patients with PM had significantly poorer overall survival than those without PM, with a median survival of 31 months in the POPM cohort (Figure 4A). Among AJCC stages, stage IVB was associated with the lowest survival (Figure 4B). Patients with a median household income exceeding $70,000 showed improved survival compared to those with an income <$70,000 (Figure 4D). Moreover, systemic therapy significantly improved survival in POPM (Figure 4E). Furthermore, early treatment (<5 days) was associated with better outcomes than delayed treatment (≥5 days) (Figure 4F). However, the T stage did not show a statistically significant effect on overall survival (Figure 4C).

No significant differences were observed in baseline characteristics between the training and validation cohorts (Table 2). Univariate Cox regression analysis identified AJCC stage IVB, median household income <$70,000, absence of systemic therapy, and time from diagnosis to treatment ≥5 days as factors associated with poorer prognosis (*p* < 0.05). Subsequent multivariate Cox regression confirmed AJCC stage, median household income, systemic therapy, and time from diagnosis to treatment as independent PFs for overall survival in POPM (Table 3).

**Table 2:** Comparison between the validation group and the training group.

Variables	Total (*n* = 212)	Validation cohort (*n* = 64)	Training cohort (*n* = 148)	Statistic	*P*
Overall survival, month, M (Q_1_, Q_3_)	21.00 (11.75, 43.25)	19.50 (9.75, 36.25)	21.50 (12.00, 44.75)	*Z* = −0.75	0.454
Median household income, *n* (%)				*χ*^2^ = 2.32	0.313
<$70,000	57 (26.89)	21 (32.81)	36 (24.32)		
$70,000–$84,999	78 (36.79)	24 (37.50)	54 (36.49)		
$85,000+	77 (36.32)	19 (29.69)	58 (39.19)		
T stage, *n* (%)				*χ*^2^ = 0.06	0.970
T1	39 (18.40)	12 (18.75)	27 (18.24)		
T2	148 (69.81)	44 (68.75)	104 (70.27)		
T3/T4/TX	25 (11.79)	8 (12.50)	17 (11.49)		
AJCC stage, *n* (%)				*χ*^2^ = 3.16	0.076
IVA	138 (65.09)	36 (56.25)	102 (68.92)		
IVB	74 (34.91)	28 (43.75)	46 (31.08)		
Systemic therapy, *n* (%)				*χ*^2^ = 0.21	0.650
No	30 (14.15)	8 (12.50)	22 (14.86)		
Yes	182 (85.85)	56 (87.50)	126 (85.14)		
Time from diagnosis to treatment in days, *n* (%)				*χ*^2^ = 1.63	0.201
<5	27 (12.74)	11 (17.19)	16 (10.81)		
≥5	185 (87.26)	53 (82.81)	132 (89.19)		
Status, *n* (%)				*χ*^2^ = 0.01	0.910
Alive	94 (44.34)	28 (43.75)	66 (44.59)		
Dead	118 (55.66)	36 (56.25)	82 (55.41)		

*Z*, Mann–Whitney test; *χ*^2^, chi-square test; M, median; Q_1_, 1st quartile; Q_3_, 3st quartile; AJCC, American Joint Committee on Cancer.

**Table 3:** Univariate and multivariable Cox proportional hazard regression analysis of overall survival.

Variables	Univariate analysis		Multivariate analysis	
	HR (95% CI)	*P*-value	HR (95% CI)	*P*-value
Median household income				
<$70,000	1.00 (Reference)		1.00 (Reference)	
$70,000–$84,999	0.51 (0.29–0.87)	0.014	0.39 (0.22–0.69)	0.001
$85,000+	0.55 (0.32–0.93)	0.027	0.43 (0.25–0.75)	0.003
T stage				
T1	1.00 (Reference)			
T2	1.28 (0.70–2.34)	0.418		
T3/T4/TX	1.92 (0.86–4.29)	0.112		
AJCC stage				
IVA	1.00 (Reference)		1.00 (Reference)	
IVB	1.69 (1.06–2.68)	0.027	1.69 (1.05–2.71)	0.030
Systemic therapy				
No	1.00 (Reference)		1.00 (Reference)	
Yes	0.37 (0.21–0.64)	<0.001	0.37 (0.21–0.65)	<0.001
Time from diagnosis to treatment in days				
<5	1.00 (Reference)		1.00 (Reference)	
≥5	2.97 (1.09–8.13)	0.034	4.27 (1.53–11.95)	0.006

HR, Hazards ratio; CI, confidence interval; AJCC: American Joint Committee on Cancer.

### 3.3 Nomogram development and confirmation

**Figure 5:**
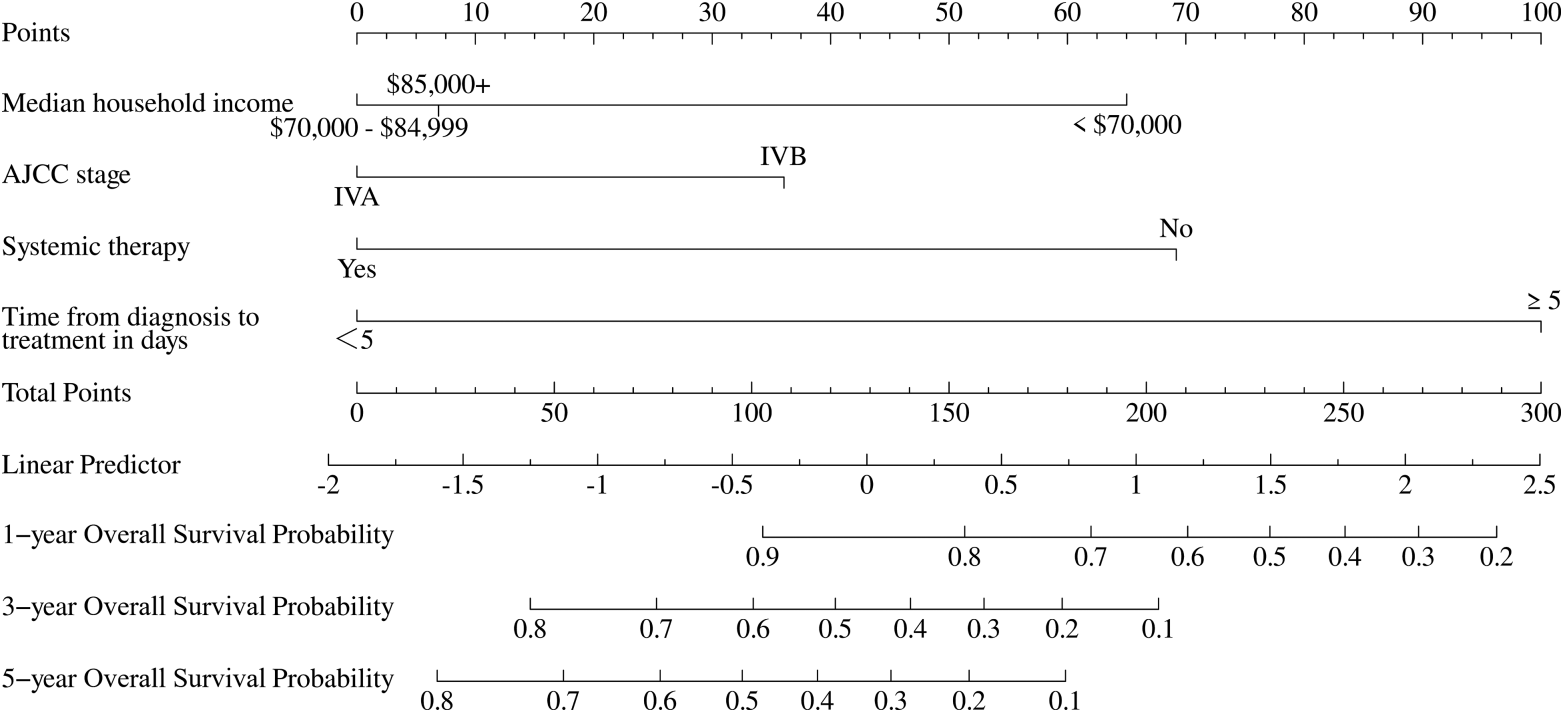
Nomogram for predicting 1-, 3-, and 5-year overall survival in pediatric osteosarcoma patients with pulmonary metastasis.

A prognostic nomogram was constructed to estimate overall survival in patients with POPM based on four independent PFs identified through multivariate Cox regression analysis (Figure 5). The nomogram demonstrated strong predictive capability, with a C-index of 0.68 (95% CI: 0.61–0.74) in the training cohort and 0.71 (95% CI: 0.62–0.80) in the validation cohort.

**Figure 6:**
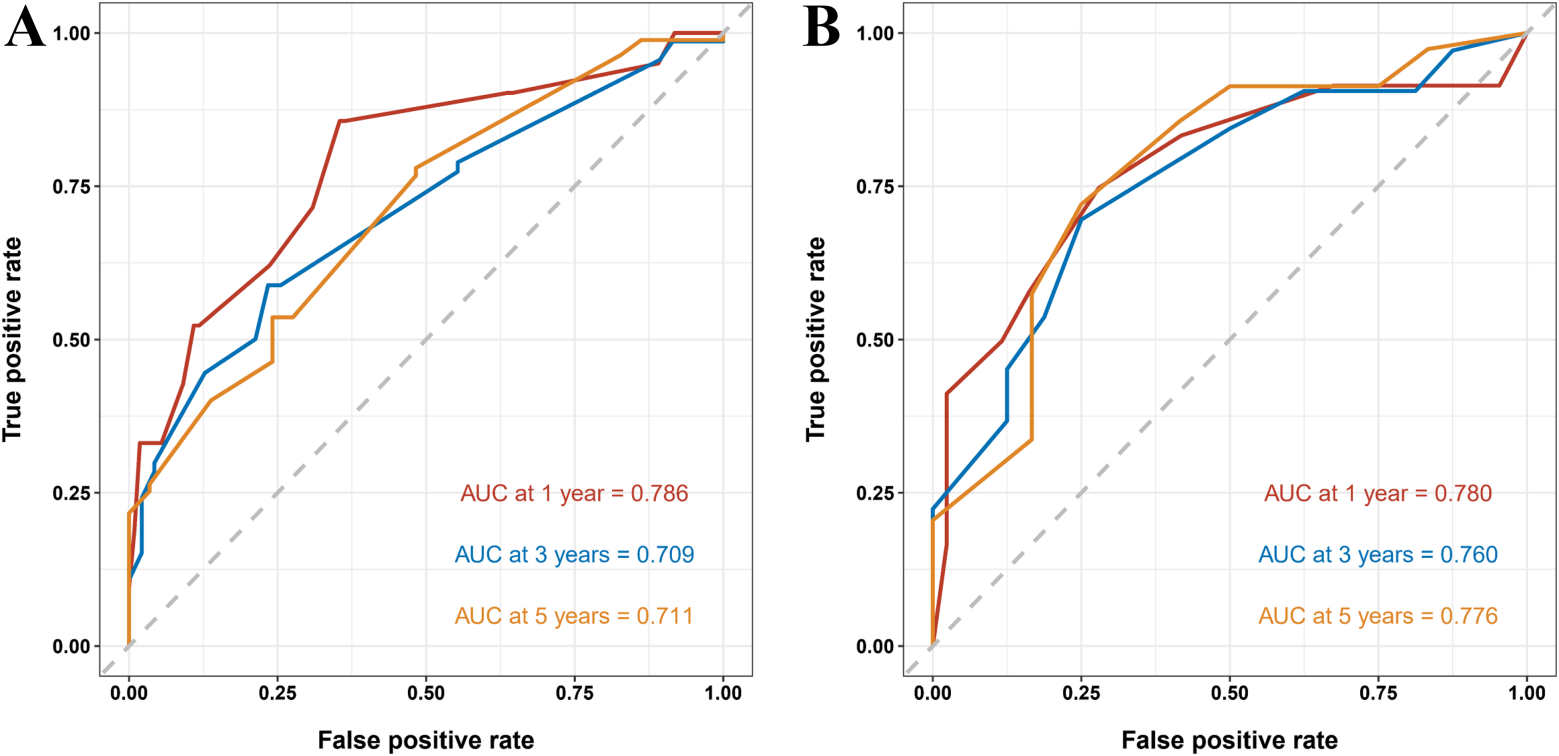
ROC curves of the nomogram predicting 1-, 3-, and 5-year overall survival in pediatric osteosarcoma patients with pulmonary metastasis. **(A)** ROC curves for the training group. **(B)** ROC curves for the validation group.

To further assess the model's predictive accuracy, ROC curves were generated for 1-, 3-, and 5-year overall survival. In the training group, the AUC values were 0.786, 0.709, and 0.711, respectively. In the validation group, AUC values were 0.780, 0.760, and 0.776, respectively (Figure 6). These results indicate strong discriminatory performance of the nomogram across different time points.

**Figure 7:**
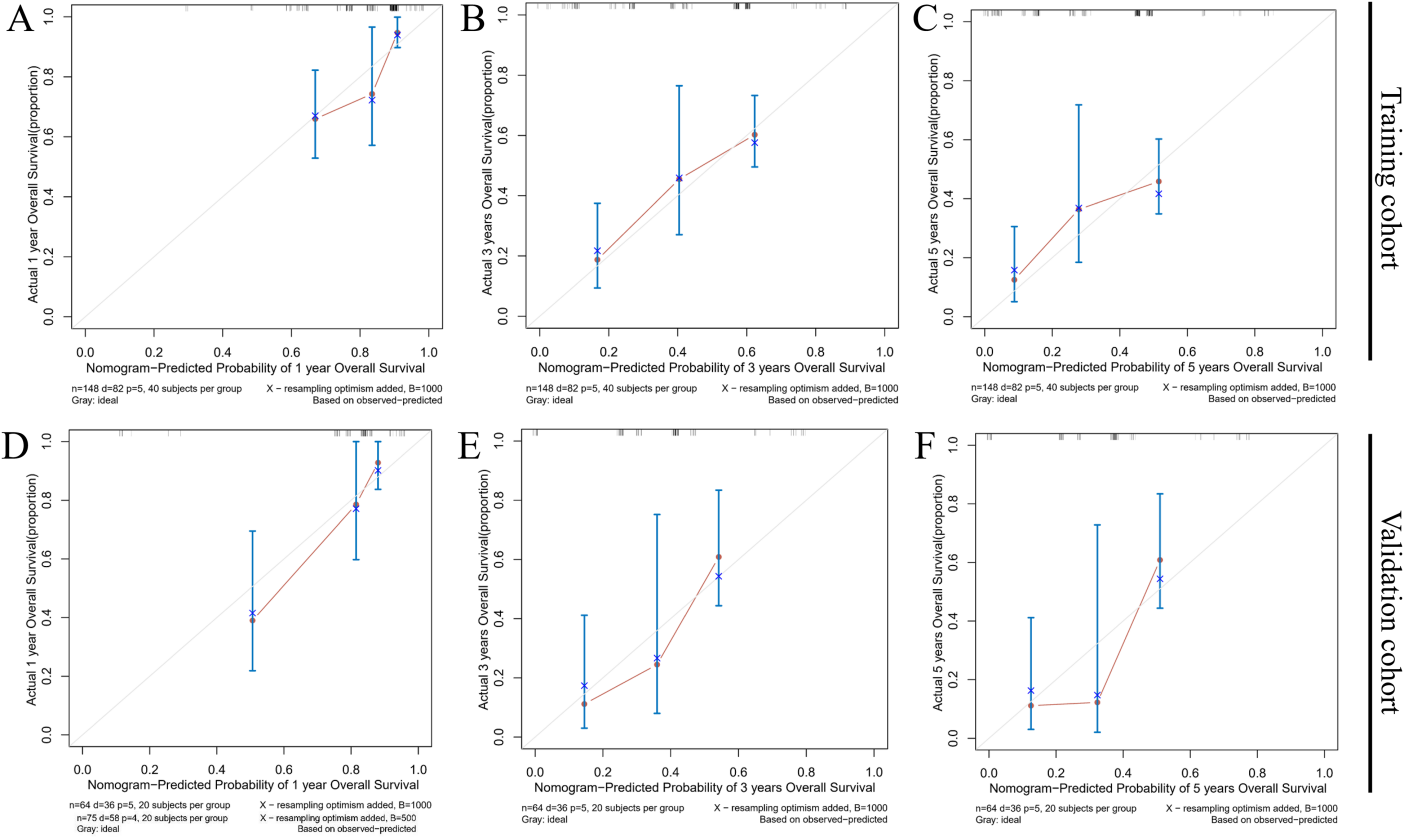
Calibration curves for the nomogram predicting 1- **(A,D)**, 3- **(B,E)**, and 5-year **(C,F)** overall survival in the training group and the validation group.

Calibration curves were used to evaluate the relationship between predicted and observed survival outcomes. The *X*-axis represented the predicted probabilities of 1-, 3-, and 5-year survival, while the *Y*-axis reflected the corresponding actual survival rates. The calibration curves displayed strong concordance between predicted and observed values, indicating high model accuracy (Figure 7).

**Figure 8:**
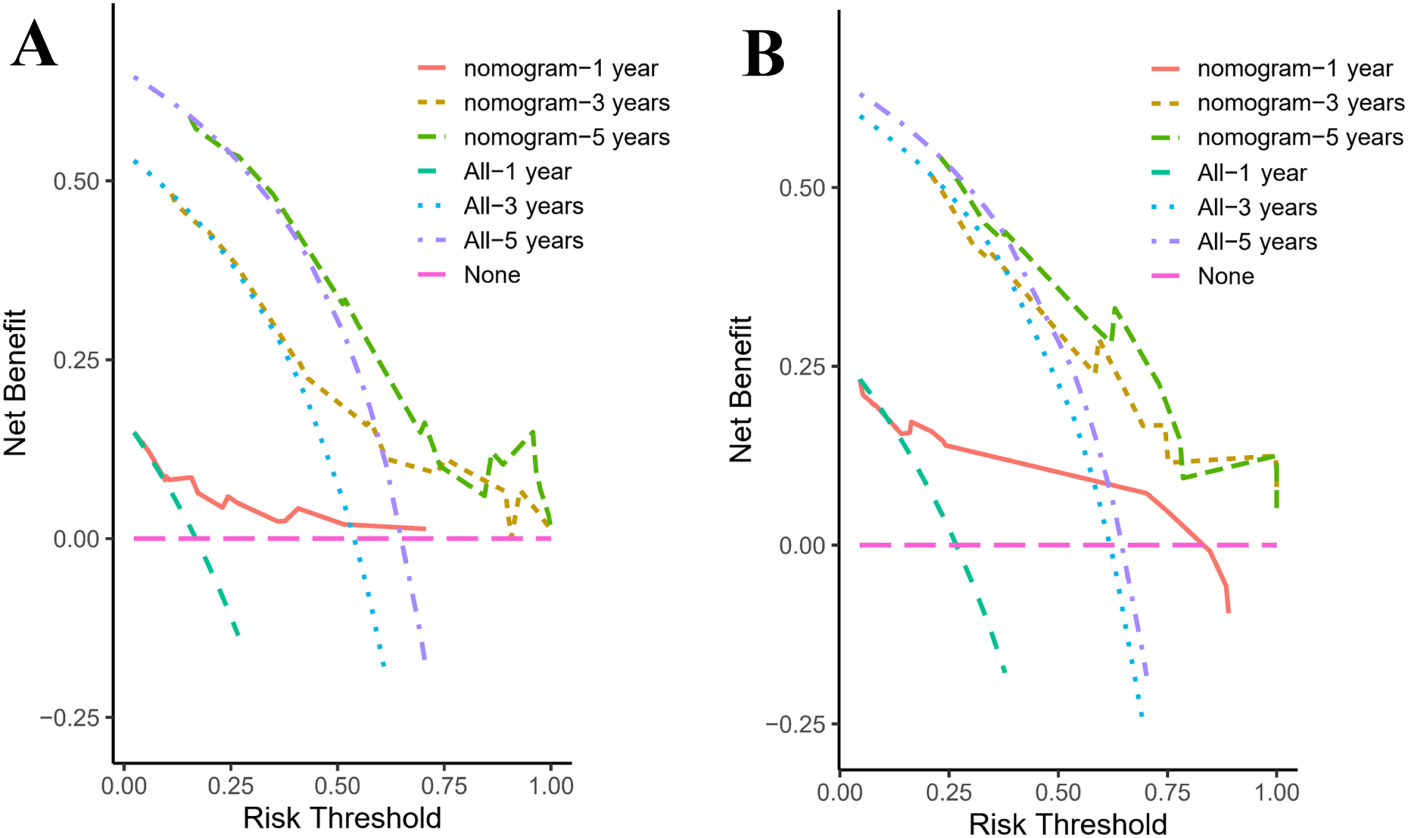
Decision curves for the nomogram predicting 1-, 3-, and 5-year overall survival in pediatric osteosarcoma patients with pulmonary metastasis. **(A)** Decision curves for the training group. **(B)** Decision curves for the validation group.

DCA was employed to assess the nomogram's clinical utility by estimating the net benefit across a range of threshold probabilities. As shown in Figures 8A,B, the nomogram yielded higher net benefits than either the treat-all or treat-none strategies across multiple thresholds, supporting its potential application in clinical decision-making.

## 4 Discussion

PM is the most common site of distant dissemination in OS and is frequently associated with poor prognosis in pediatric patients. Several studies have identified risk factors for PM in OS, including age, tumor stage, grade, size, bone metastasis, N stage, and axial tumor location (12, 16–19). Consistent with previous findings, the present analysis of 1,162 pediatric OS cases from the SEER database confirmed that tumor size, T stage, and N stage are significant risk factors for PM. However, research specifically focused on the prognosis of POPM remains limited. Zongtai Liu et al. investigated 114 pediatric and adolescent OS cases with PM from the SEER database and constructed a prognostic nomogram using Cox regression. Their model identified independent prognostic factors such as age, surgical intervention, chemotherapy, primary tumor site, and bone metastasis (12). On the other hand, the current study analyzed a larger cohort of 212 pediatric OS cases with PM diagnosed between 2010 and 2021 and incorporated more recent clinical variables. Prognostic factors, including AJCC stage, T stage, systemic therapy, median household income, and time from diagnosis to treatment, were identified using LASSO regression, and their independent prognostic value was confirmed via multivariate Cox regression. The nomogram developed in this study demonstrated improved accuracy and calibration compared with the model proposed by Liu et al., providing more effective guidance for clinical decision-making and potentially improving patient outcomes.

The findings from this study highlight the importance of AJCC stage, systemic therapy, median household income, and time from diagnosis to treatment (days) as distinct PFs for POPM. The AJCC staging system, a widely recognized diagnostic and prognostic system, is essential in guiding treatment decisions. Higher AJCC stages are associated with worse survival outcomes in gallbladder cancer, breast cancer, and colorectal cancer (20–22). Our investigation revealed that regarding the AJCC stage, 138(65.09%) cases were classified as AJCC stage IV A [Any T, N0, M1a (lung metastasis), any histologic grade]. There were 74 (34.91%) patients with AJCC stage IV B [Any T, N1 (regional lymph node metastasis) or M1b (other distant sites), any histologic grade]. Stage IVB POPM patients exhibited significantly worse prognosis. Similarly, the advanced AJCC stage in OS patients also demonstrated a close association with poorer survival (11, 23). This study highlights the importance of accurate staging in guiding treatment decisions and estimating prognosis for POPM. While the T stage emerged as a significant prognostic factor in LASSO regression analysis, its lack of statistical significance in subsequent univariate and multivariate Cox regression analyses warrants further exploration. This discrepancy may stem from the fact that POPM represents an advanced metastatic malignancy, whereas T staging predominantly reflects the biological characteristics of local tumor invasion in primary lesions.

Median household income, a key socioeconomic variable, emerged as an independent prognostic factor for POPM in this study. Patients from higher-income households (>$70,000) showed significantly improved survival compared to those from lower-income households (<$70,000). This association can be interpreted from multiple perspectives. Higher-income families typically have better access to advanced medical technologies, specialized healthcare teams, and comprehensive treatment services, all of which contribute to better clinical outcomes (24, 25). Similarly, improved healthcare quality may reduce treatment complications and improve survival. Nutritional status, which often correlates with socioeconomic status, also plays a key role in supporting recovery during and after therapy. Furthermore, families with higher incomes may provide stronger psychological and social support systems, which can reduce treatment-related psychological stress (26). The observed association between household income and POPM prognosis highlights the systemic nature of healthcare inequality and its significant effects on clinical outcomes. This association is likely driven by complex socioeconomic determinants, including differential access to medical resources, variability in treatment adherence, and disparities in support systems across income groups. Addressing these inequities is essential for improving the prognosis of POPM. This finding highlights the importance of integrating socioeconomic variables, alongside clinical and biological factors, into treatment planning and risk stratification. While the precise mechanisms underlying this association require further investigation, it is clear that socioeconomic factors significantly affect healthcare access, treatment quality, and overall disease management. Systemic therapy, which targets cancer cells throughout the body, remains a cornerstone of OS treatment and primarily involves chemotherapy, with emerging roles for targeted therapy and immunotherapy (27). Multidrug chemotherapy remains the primary systemic approach for OS management (28, 29). In this study, the administration of systemic therapy was strongly associated with improved overall survival. Patients who received systemic therapy experienced significantly longer survival than those who did not. These findings align with previous research showing the survival benefits of chemotherapy in OS (30) and further extend this observation to the POPM population. Emerging therapies, including molecularly targeted agents and immunotherapeutic strategies, are being explored for OS and may offer more personalized and effective treatment modalities (31–33). These results highlight the clinical efficacy of systemic chemotherapy in managing both primary and metastatic disease, slowing disease progression, and improving survival outcomes. However, there remains an urgent need to develop novel treatment strategies that further improve overall survival in POPM.

Time from diagnosis to treatment also emerged as a key PF for POPM in this study. Consistent with observations from other malignancies, treatment delays have been shown to negatively affect survival outcomes. Previous studies have reported that even a 4-week delay in initiating treatment is associated with increased mortality risk (34), highlighting the clinical importance of timely intervention. In this study, POPM patients who experienced treatment delays of more than 5 days had significantly poor survival outcomes. This effect may be explained by accelerated tumor progression or the development of multisystem complications during the treatment interval, particularly given the aggressive behavior often seen in early-stage POPM. These findings highlight the need for prompt therapeutic intervention and suggest that minimizing delays between diagnosis and treatment could improve prognosis. Understanding the underlying mechanisms by which even a short treatment delay (> 5 days) contributes to poor prognosis in POPM remains an important focus for future research.

Despite the strengths of this study, several limitations must be considered. First, essential clinical variables were not available in the SEER database, including (1) details of chemotherapy regimens, (2) histological response to neoadjuvant chemotherapy, and (3) molecular profiling data. Second, limited racial diversity was observed within the study population, with Caucasian patients accounting for 74.06% of the POPM cohort, while African American and other racial groups were underrepresented. This demographic imbalance may restrict the generalizability of the findings across diverse populations. Third, although the nomogram demonstrated strong performance in internal validation, external validation using independent datasets remains necessary to confirm its reliability across diverse clinical settings. Future research should aim to (1) incorporate comprehensive chemotherapy-related data from institutional registries, (2) conduct multicenter studies to increase demographic representation, and (3) employ advanced causal inference approaches, such as target trial emulation, to reduce confounding from unmeasured variables.

## 5 Conclusion

In summary, this study developed a novel nomogram to predict overall survival in POPM patients based on a cohort of 212 cases from the SEER database. The model demonstrated robust predictive performance and may serve as a valuable clinical tool to support personalized survival predictions, guide therapeutic decision-making, and improve follow-up planning in this high-risk population.

## Data Availability

The original contributions presented in the study are included in the article/Supplementary Material, further inquiries can be directed to the corresponding author.
